# Comparative Transcriptomic Analysis of Two Bottle Gourd Accessions Differing in Fruit Size

**DOI:** 10.3390/genes11040359

**Published:** 2020-03-27

**Authors:** Hongyuan Zhang, Jie Tan, Min Zhang, Shuping Huang, Xia Chen

**Affiliations:** Wuhan Academy of Agricultural Sciences, Wuhan 430070, China; hongyuanzhang2011@163.com (H.Z.); aggas@126.com (J.T.); zhangmin818@aliyun.com (M.Z.); hsping80@163.com (S.H.)

**Keywords:** *Lagenaria siceraria*, candidate genes, fruit size, transcription factors, gene expression

## Abstract

The bottle gourd (*Lagenaria siceraria*) is an important horticultural and medicinal crop with high nutritional value. This study aimed at examining the molecular regulation of fruit size in bottle gourd. We performed transcriptome sequencing of two bottle gourd cultivars differing in their fruit size. The average fruit length and weight of the cultivar Hang (39.48 cm/624.4 g) were higher than those of the cultivar USA (10.34 cm/152.8 g) at maturity. Transcriptome sequencing and assembly resulted in 89,347 unigenes. A total of 1250 differentially expressed genes (DEG) were found between the two cultivars, including 422 upregulated genes and 828 downregulated genes in Hang as compared to USA. Genes related to cell wall metabolism, phytohormones, cell cycle, and cell division showed significant differential expression between the two cultivars. DEGs encoding transcription factors (TF) from nine TF families were also identified. The ethylene response factor family was the most enriched among these families. Our study provides a basis for further investigations of the molecular regulation of fruit size in bottle gourd.

## 1. Introduction

The bottle gourd (*Lagenaria siceraria*, 2n = 2× = 22), a member of the Cucurbitaceae family [1], is an important horticultural and medicinal crop [2]. It originated in Africa and was independently domesticated in Africa and Asia [3]. The fruit of bottle gourd can be used as vegetable, utensil, ornamental, and even as a musical instrument. The bottle gourd is a great source of essential nutrients, such as vitamin B complex, pectin, fibers, ascorbic acid, beta-carotene, protein, amino acids, and minerals [4].

Bottle gourd fruit varies in shape and size [5,6]. Various genetic components are involved in determining fruit size and shape during fruit development [7]. Fruit development in fleshy fruits can generally be divided into two stages, i.e., early fruit development and fruit ripening. Early fruit development involves (i) fruit setting, (ii) fruit growth by cell division, and (iii) fruit growth by cell expansion until it reaches its final size and shape [8]. Rapid cell division and cell elongation occur during the growth stage and determine the final size and shape of the fruit [9]. Fruit size and shape are among the most important characters of any fruit which determine its market value. Optimization of desired shape and size of the fruit demands extensive investigation of molecular mechanisms regulating fruit development.

The plant cell wall is mainly composed of polysaccharides. Hundreds of genes are involved in cell wall metabolism [10]. Regulation of cell wall biogenesis and modification plays an important role in cell expansion and unidirectional elongation during fruit development [11,12]. Expansins are some of the most well characterized cell wall proteins that regulate cell size and growth during all plant growth stages and fruit development [13]. Previous studies showed that expansins are abundantly expressed during fruit development and ripening [14]. The cell cycle regulation is of key importance for plant growth and development. Cell division and expansion are controlled by various factors. Cyclins and cyclin-dependent protein kinases are the main regulators of the cell cycle [15]. Many studies have shown an increase in cyclins’ expression during early fruit development in tomato and cucumber [14,16,17].

Hormones also contribute to regulating fruit development and size [18]. The seeds are rich in auxin and cytokinin, which are the main regulators of fruit development and also determine the fruit size. An increase in auxin and cytokinin levels in seeds is correlated with fruit growth stages [19]. The role of auxin in cell expansion during fruit development stages is well established [20,21]. Numerous studies have demonstrated the role of auxin response factors (ARFs), the main regulators of auxin, in cell division and growth during fruit development [20,21,22,23,24]. Transcription factors also regulate genes related to fruit development [8,25,26].

Recently, transcriptomic sequencing has been used to study the molecular basis of fruit development in many fleshy fruits, including cucumber, melon, and tomato [14,27,28,29,30,31]. However, most of the studies focused on fruit ripening and quality, and very few specifically investigated genes related to fruit size, at least in Cucurbitaceae species. Qi et al. [32] identified a candidate gene that encodes cyclins controlling cell proliferation and fruit size in cucumber and *C. sativus*. Jiang et al. [15] also highlighted the important role of microtubules and cyclins-related genes in determining cucumber fruit size. Later on, Wang et al. [33] pinpointed the key role of auxin and cytokinin signaling in fruit length of cucumber. All these previous studies suggest that phytohormones, cell wall metabolism, and cell cycle and cell division are the key pathways for fruit size determination in Cucurbitaceae.

In the present study, we investigated the transcriptome of two cultivars of bottle gourd (USA and Hang) with contrasting fruit size. Our goal was to identify key pathways, structural genes, and regulators involved in the differential fruit size. It is anticipated that the information generated in this work will be helpful for bottle gourd breeding programs aimed at developing cultivars with improved fruit size.

## 2. Materials and Methods

### 2.1. Plant Materials

Two cultivars of bottle gourd (*Lagenaria siceraria*, 2n = 2× = 22 cv. Hang and USA) were used in this study. These commercial cultivars are highly grown and consumed in Wuhan, China. Fruit size of USA is considerably shorter than that of Hang. The plants were grown in the greenhouse of the Vegetable Institute of Wuhan Academy of Agricultural Sciences, Wuhan, China. Plants were grown in a substrate composed of vermiculite:perlite: organic fertilizer = 4:4:1:1. The pH of the culture substrate was about 7.0, and the water content was maintained at about 70% relative humidity. The greenhouse conditions were maintained at 25–28/18–20 °C day/night temperature, photoperiod 14 h/d with a light intensity of 87.5 µmol m^−2^ s^−1^. Pest control was performed according to standard practices. Mature fruits were harvested from three individual plants of each cultivar, and 2 cm^3^ of the pericarp was cut, immediately frozen in liquid nitrogen, and stored at −80 °C until further use.

### 2.2. Histological Analysis

A scalpel was used to cut the pericarp tissue into thin slices. The slices were fixed for 12 h in Carnoy’s fixative solution (alcohol, acetic acid; V:V 3:1), stained 2 min with 1% Janus green, and then rinsed with 70% ethanol for 10 sec. Sections were examined using an Olympus BX-50 microscope.

### 2.3. RNA Extraction and Quality Test

Six frozen fruit samples were ground in a mortar with liquid nitrogen, and total RNA was isolated using the RNA extraction kit (Huayueyang, China). RNA degradation and contamination were monitored on 1% agarose gels. RNA purity was checked using the NanoPhotometer^®^ spectrophotometer (IMPLEN, CA, USA). RNA integrity was assessed using the RNA Nano 6000 Assay Kit of the Agilent Bioanalyzer 2100 system (Agilent Technologies, CA, USA).

### 2.4. RNA-Seq Library Preparation and Sequencing

A total amount of 3 μg RNA per sample was used as input material for the RNA sample preparations. Sequencing libraries were generated using NEBNext^®^Ultra™ RNA Library Prep Kit for Illumina^®^ (NEB, Carlsbad, CA, USA) following the manufacturer’s recommendations, and index codes were added to attribute sequences to each sample. Briefly, mRNA was purified from total RNA using poly-T oligo-attached magnetic beads. Fragmentation was carried out using divalent cations under elevated temperature in NEBNext First Strand Synthesis Reaction Buffer (5×). First strand cDNA was synthesized using random hexamer primers and M-MuLV Reverse Transcripta (RNase H^−^). Second strand cDNA synthesis was subsequently synthesized using DNA Polymerase I and RNase H. Remaining overhangs were converted into blunt ends via exonuclease/polymerase activities. After adenylation of the 3′ ends of DNA fragments, NEBNext Adaptors with a hairpin loop structure were ligated to prepare for hybridization. To select cDNA fragments of 150~200 bp in length preferentially, the library fragments were purified with AMPure XP system (Beckman Coulter, Beverly, MA, USA). Then 3 μl USER Enzyme (NEB, Carlsbad, CA, USA) was used with size-selected, adaptor-ligated cDNA at 37 °C for 15 min followed by 5 min at 95 °C before PCR. Then PCR was performed with Phusion High-Fidelity DNA polymerase, Universal PCR primers, and Index (X) Primer. At last, PCR products were purified (AMPure XP system), and library quality was assessed on the Agilent Bioanalyzer 2100 system. The clustering of the index-coded samples was performed on a cBot Cluster Generation System using a TruSeq PE Cluster Kit v3-cBot-HS (Illumina, San Diego, CA, USA) according to the manufacturer’s instructions. After cluster generation, the library preparations were sequenced on an Illumina Hiseq 2000 platform, and paired-end reads were generated.

### 2.5. Quality Control and Transcriptome Assembly

Raw data of fastq format were first processed through in-house perl scripts. In this step, clean data were obtained by removing reads containing adapters, reads containing poly-N, and low-quality reads from raw data. At the same time, Q20, Q30, GC-content, and sequence duplication level of the clean data were calculated. All the downstream analyses were based on clean data with high quality. Transcriptome assembly was accomplished using Trinity [34] with min_kmer_cov set to 2 by default, and all other parameters set to default.

### 2.6. Quantification of Gene Expression and Differential Expression Analysis

Gene expression levels were estimated by RSEM [35] for each sample. Clean data were mapped back onto the assembled transcriptome. The read count for each gene was obtained from the mapping results. Differential expression analysis was performed using the DESeq R package (1.10.1) [36]. *p*-value was adjusted using *q*-value [37]. *q*-value < 0.005 and |log2(fold change)| >1 was set as the threshold for significantly differential expression.

### 2.7. GO and KEGG Pathway Enrichment Analysis

Gene ontology (GO) enrichment analysis of the differentially expressed genes (DEGs) was implemented by the topGO R packages based Kolmogorov–Smirnov test. We used KOBAS [38] software to test the statistical enrichment of differential expression genes in Kyoto Encyclopedia of Genes and Genomes (KEGG) pathways.

### 2.8. Validation of RNA-Seq Results by qRT-PCR

Total RNA was extracted from the flesh of bottle gourd USA and Hang at the mature stage. RNA-free DNaseI (Fermentas, Waltham, MA, USA) was used to remove DNA contamination for 20 min at 37 °C. Approximately 1 μg of total RNA was reverse transcribed using Oligo(dT)_18_ and a Fermentas Revert Aid First Strand cDNA Synthesis Kit (Fermentas, Glen Burnie, MD, USA). The reactions were incubated for 30 min at 16 °C, followed by 60 cycles of pulsed reverse transcription at 30 °C for 30 s, 42 °C for 30 s and 50 °C for 1 s; and finally terminated by incubating at 70 °C for 5 min. Relative expression analysis of the mRNA was performed using the ABI Step One Plus™ Real Time PCR System (Applied Biosystems, Carlsbad, CA, USA) and SYBR Green Master Mix (Roche, Berlin, Germany). Quantitative real-time PCR was performed using the following parameters: 5 min at 95 °C, followed by 40 cycles of 15 s at 95 °C and 60 s at 60 °C. *LsH3* was chosen as the endogenous control [39]. The reactions were performed with three biological replicates, and a melting curve analysis was carried out to verify that only one specific amplification occurred. Comparative expression levels were calculated in the four different samples using the 2^−ΔΔCT^ method. Primer sequences are provided in Table S1.

## 3. Results

### 3.1. Overview of the Transcriptome Sequencing

**Figure 1:**
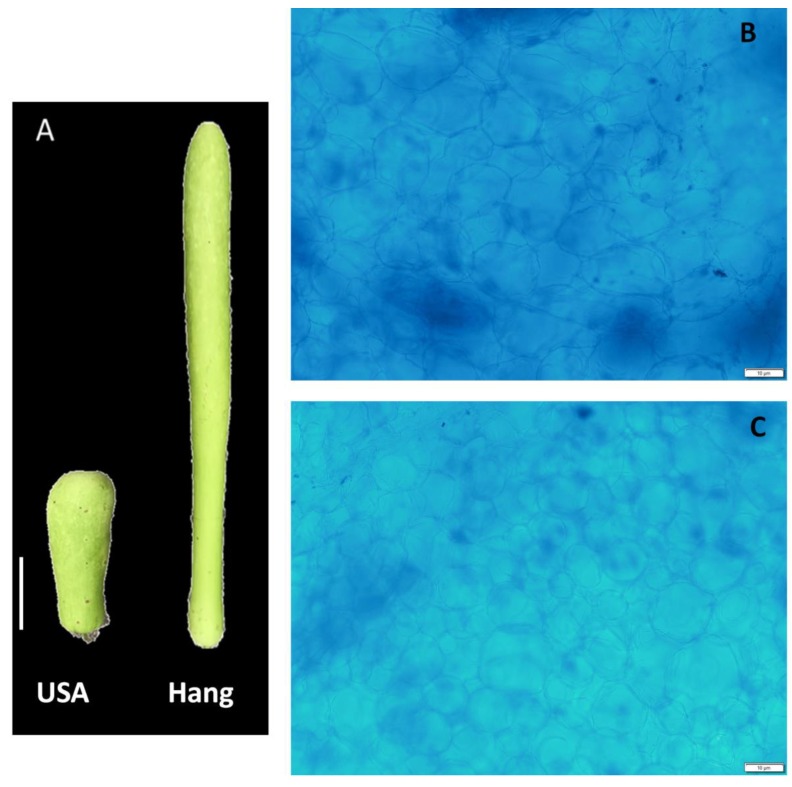
Phenotypes of the bottle gourd fruit Hang and USA. (**A**) Pictures of fruits of the two cultivars at maturity; bar = 5 cm (**B**) Histological photograph of pericarp section in Hang fruit; (**C**) Histological photograph of pericarp section in USA fruit; bar = 10 µm.

We performed transcriptome sequencing of two bottle gourd cultivars with varying fruit sizes (Figure 1A). Fruit length and weight were measured at the marketable mature fruit stage. The average fruit length of Hang and USA was 39.48 ± 2.30 cm and 10.34 ± 1.49 cm, respectively. Similarly, the average fruit weight was 624.4 ± 19.81 g and 152.8 ± 10.36 g for Hang and USA, respectively. We further performed histological analyses of the pericarp to observe cell size and cell number. From the pictures, it was noticed that Hang cells (Figure 1B) were slightly larger than USA cells (Figure 1C), while no obvious change was observed for the cell number. We deduce that variation in cell size is the underlying reason for the difference in Hang and USA fruit sizes. Nonetheless, since our histology analysis was mainly descriptive, a new experiment based on a quantitative approach is required to assess whether there is no significant difference in cell numbers between the two cultivars.

Six sequencing libraries (three repeats for each cultivar) were constructed. After sequencing quality control, a total of 43.60 GB clean data (with an average of 7.20 GB per sample) was obtained, and the percentage of Q30 score of all products was not less than 92.52% (Table 1). A total of 89,347 Unigenes were obtained by assembly. The N50 of Unigenes was 1318 bp, with high assembly integrity (Figure S1; Table 2).

### 3.2. Gene Expression Analysis

**Figure 2:**
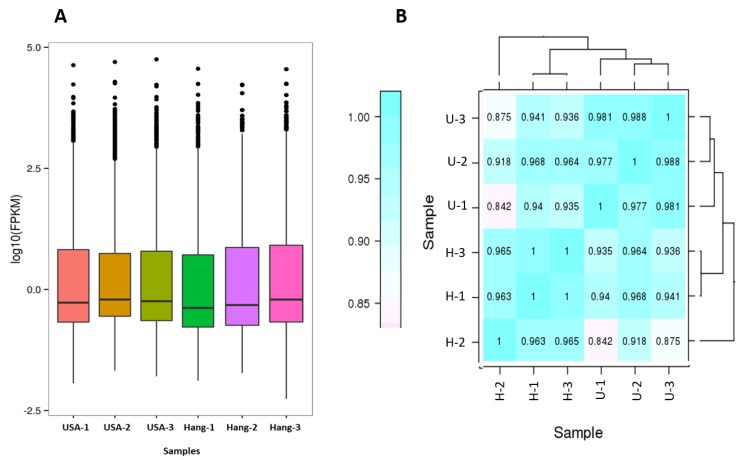
Overview of the transcriptome sequencing. (**A**) Gene expression profiles in the six libraries; (**B**) Heatmap clustering showing correlation among samples based on global expression profiles. U represents the USA fruit, while H represents the Hang fruit.

**Figure 3:**
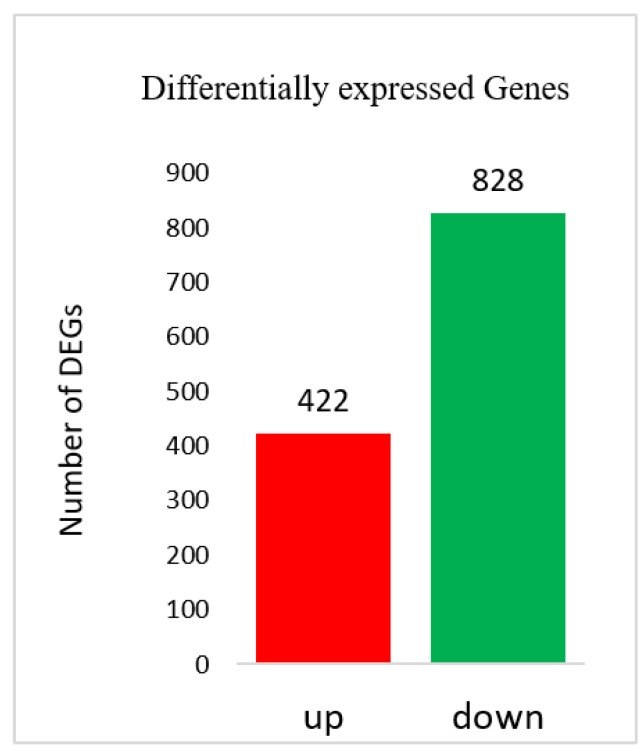
Differentially expressed genes (DEGs) identified between the two accessions.

Fragments per kilobase per million mapped reads (FPKM) values were used to calculate the expression abundance of corresponding unigenes. Overview of transcriptome sequencing is shown in Figure 2. Overall, similar global gene expression could be observed among samples, and Pearson’s correlations between replicates ranged from 0.842 to 0.988 (Figure 2). A total of 1250 differentially expressed genes (DEG) were found between USA and Hang. Downregulated DEGs outnumbered the upregulated genes (Figure 3; Table S2).

### 3.3. Bottle Gourd Fruit Size Related DEGs

#### 3.3.1. Cell Wall Metabolism

Rapid cell elongation occurs during all plant growth stages [40]. The modification of the plant cell wall is highly required for the release of wall stress and a decrease in rigidity to allow cell elongation [12,41,42]. We identified 19 cell wall metabolism-related genes showing significant differential expression. Among these, 13 DEGs had higher expression in Hang than USA. Xyloglucans are hemicelluloses present in the cell wall and are required for cell wall organization [43]. We identified four DEGs related to xyloglucan metabolism. Two of these DEGs were upregulated in Hang. Expansins are also very important for the loosening of the cell wall to allow cell elongation and growth [11,13]. Four expansin genes were significantly upregulated in Hang. Endoglucanases are a fundamental part of the cell wall metabolic process [10,44]. Six DEGs related to endoglucanases showed significantly differential expression in fruit development. Five of them were upregulated in Hang (Table 3).

#### 3.3.2. Cell Cycle and Cell Division

Regulation of cell cycle and cell division directly affects the growth rate of plant tissues and also determines the final size of plant organs [19,40]. We identified six DEGs involved in the regulation of cell cycle and cell division. Four of these DEGs were upregulated in Hang fruits (Table 4).

#### 3.3.3. Regulation of Hormones Related to Fruit Size in Bottle Gourd

The role of plant hormones in the regulation of plant growth and fruit development is well established [18]. Auxin plays a role in cell elongation during fruit development stages [12,18]. We identified six DEGs related to auxin. Four of these were highly upregulated in Hang. Cytokinin regulates cell division during fruit development [19]. In our study, three cytokinin genes showed significant differential expression. One of these was upregulated in Hang (Table 5). Gibberellin regulates plant growth and development at various stages. We found one gibberellin 3-β-dioxygenase 1 gene, which was downregulated in Hang fruit (Table 5).

#### 3.3.4. Analysis of Homologs of Well-Known Genes Involved in Fruit Size in Bottle Gourd

In other plants, such as tomato and *Arabidopsis thaliana*, some genes have been characterized, and their functions in controlling fruit size have been demonstrated. In our DEGs, we detected the gene *c25755.graph_c0,* which is the homolog of *fruit weight 2.2* (*FW2.2*) from tomato [48]. The gene *c25755.graph_c0* was 16-fold higher expressed in USA than in Hang fruit. We also identified *c52330.graph_c0* as the homolog of *Cell Size Regulator* (*CSR*) from tomato [49]. The expression level of *c52330.graph_c0* was six-fold higher in Hang than USA. Furthermore, *c39771.graph_c0* is the homolog of *AtHXK-1* known to regulate fruit size in Arabidopsis [50]. Our data showed that *c39771.graph_c0* was upregulated in USA, indicating a negative role in fruit size in bottle gourd.

#### 3.3.5. Transcriptional Regulation of Fruit Size in Bottle Gourd

Transcription factors (TF) regulate gene expression during all developmental and growth stages of the plant, leaving no exception for fruit development [8]. We identified 26 DEGs from eight TF families showing significant differential expression during fruit development. The ethylene-responsive transcription factor (ERF) family had the highest number of DEGs. All of the ERFs were highly upregulated in Hang. Four members, each of WRKY and bHLH families, showed significant differential expression (Table 6).

### 3.4. Validation of Fruit Size Related Genes by qRT-PCR

**Figure 4:**
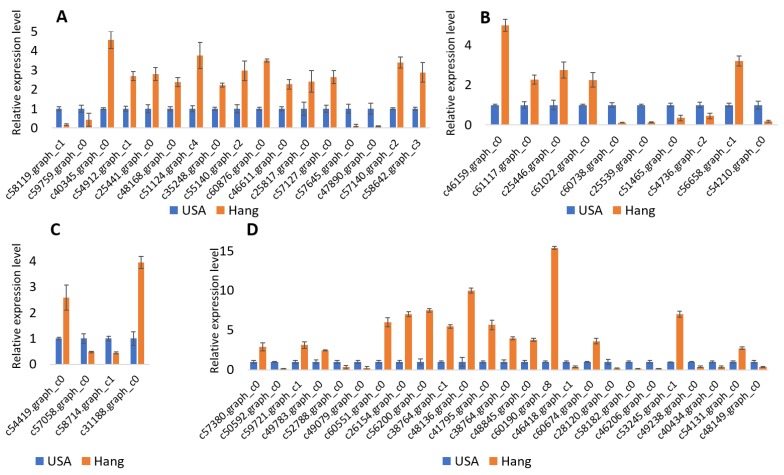
qRT-PCR results of genes related to bottle gourd fruit size. (**A**) Genes related to cell wall metabolism during fruit development; (**B**) Genes related to hormones involved in fruit development; (**C**) Genes controlling cell cycle and cell division during fruit development; (**D**) Transcription factors regulating fruit size.

We further performed qRT-PCR analysis to validate some candidate genes related to bottle gourd fruit size. As shown in Figure 4, there were significant differences in gene expression between the two accessions, and the trend perfectly matched observations in the RNA-seq. These results support the reliability of our RNA-seq report and subsequent interpretations.

## 4. Discussion

Transcriptomic sequencing of two bottle gourd accessions yielded 89,347 Unigenes. A small fraction of these Unigenes showed differential expression for bottle gourd fruit size, which indicates that few genes related to bottle gourd fruit size were expressed at the maturity stage. The mining of differentially expressed genes (DEGs) related to fruit size could be improved by sampling at early growth stages of bottle gourd fruit, and this is the objective of an ongoing study.

Fruit growth is a result of rapid cell division and cell elongation until the fruit gains its maximum size and starts ripening [40]. Cyclins are well known for regulating cell cycle [51]. The cell cycle is regulated at two points, G1/S and G2/M transition phases [52]. Cyclins, along with cyclin-dependent kinases, regulate cell cycle at these two checkpoints by phosphorylation of excessive substrate, thus starting DNA replication and mitosis [53]. We identified three cyclin genes showing significantly higher expression in the big size fruits of Hang cultivar. *c58714.graph_c1* encodes G2/mitotic-specific cyclin-1, which is essential for the regulation of the G2/M phase transition in mitosis [54]. *c54419.graph_c0* encodes Cyclin-D4-1, which is involved in the G1/S phase transition during cell division [55]. Higher expression levels of these cyclin genes in Hang fruits suggest their positive role in fruit size determination.

The modification of the plant cell wall is highly required for the release of wall stress and a decrease in rigidity to allow cell elongation. Expansins are known for the modification of the cell wall during the growth stage. Expansins cause extension in the cell wall by disrupting the non-covalent bond between cell wall microfibrils and glucan matrix [56]. Plant tissues under rapid growth are supposed to have a higher level of expansin gene expression [57]. Our findings supported this idea. Four expansin genes showing significant differential expression were identified. All of these genes were highly upregulated in Hang fruits, which suggests that these genes could be important candidates for increasing bottle gourd fruit size. Further validation and characterization of these genes can be done by transgenic approaches. Endoglucanases are involved in endohydrolysis of (1->4)-β-D-glucosidic linkages in cellulose and contribute to cellulose microfibril formation and cell wall organization during elongation. Six DEGs related to endoglucanase were identified. Five of these were upregulated in Hang fruits. Xyloglucans are an essential component of the cell wall. Xyloglucan endotransglucosylase/hydrolases (XTH) cleaves and relegates xyloglucan polymers and participates in cell wall construction in growing tissues. Four XTH genes were differentially regulated in the two cultivars, including up- and downregulated genes. Recently, Han et al. [58] found that two members *DkXTH6* and *DkXTH7* of the XTH family, have opposite expression patterns and play special and divergent roles in persimmon fruit. While one takes part in cell wall restructuring, the other is involved in cell wall assembly. In this study, differential expression of cell wall modification-related genes between the two bottle gourd cultivars suggests their active role in regulating the fruit size in bottle gourd. Further investigations will be required to elucidate the specific function of each DEG and their impact on fruit size.

Hormones also contribute to the regulation of fruit development. Cytokinin accelerates cell division during the growth stage [59]. Cytokinin dehydrogenase (CKX) breaks down the cytokinin [60]. Overexpression of cytokinin dehydrogenase in Arabidopsis led to alterations in plant growth [61]. Two CKX genes were downregulated in Hang fruits suggesting that these genes negatively regulate fruit size. The role of auxin in plant cell expansion is well established [20]. Many studies have demonstrated the role of auxin response factors (ARFs), the main regulators of auxin, in cell division and plant growth [21,23,24,62]. We identified eight auxin-related genes showing significant differential expression among the two cultivars. These genes represent important resources to be further characterized to uncover their specific roles in the regulation of fruit size in bottle gourd.

Although several studies were conducted to identify quantitative trait loci related to fruit size and shape in bottle gourd [63], no putative gene has been specifically reported so far. Well characterized genes related to fruit size were identified in other plants, such as tomato and Arabidopsis. Based on our transcriptome assembly, we successfully identified several homologs in bottle gourd. Fruit weight 2.2 is a negative regulator of cell division and determines fruit size in tomato [48]. In our study, the expression of the homologous gene was significantly repressed in Hang. In accordance with the notion that Cell Size Regulator (*CSR*) positively controls cell size and fruit weight, we observed an upregulation of *CSR* in Hang [49]. Furthermore, *AtHXK-1* activity has been shown to perturb carbon and energy metabolism and reduce fruit and seed size in tomato [50]. The homologous gene found within our DEGs showed an upregulation in the small-size fruits of USA. Therefore, we deduce that the molecular mechanisms related to fruit size in other well studied plants may be conserved in bottle gourd, and the homology-based candidate gene mining could be an efficient way to pinpoint important gene resources [64].

Regulation of the expression levels of structural genes is under the tight control of transcription factor (TF) [8]. In this study, the upregulation of several ERFs in Hang fruit denotes that they positively contribute to the big fruit size. Numerous studies identified ERF genes as master regulators of the expression levels of phytohormones, cell wall modification, cell cycle, and other important structural genes involved in fruit development and ripening [65,66,67]. Decoding the networks connecting ERFs and other TFs identified in the present work to their target structural genes will highlight the most important genes to target for efficient fruit size manipulation in bottle gourd [68].

## 5. Conclusions

We performed RNA-seq of two bottle gourd accessions to study the genes controlling the fruit size. Although mature fruits were used in this study, we successfully identified several candidate genes related to cell wall metabolism, phytohormones, cell division, cell cycle regulation, and transcription factors that probably regulate fruit size in bottle gourd. Sampling at different time points during fruit growth will provide a better insight into the molecular mechanisms of bottle gourd fruit development and size. Overall, our study provides a basis for further investigation of the molecular regulation of fruit growth and size in bottle gourd.

## Figures and Tables

**Table 1:** Sequencing data output statistics.

Sample	Reads Number	Base Number	GC (%)	Q30 (%)
USA-1	24,654,704	7,324,872,010	46.44	95.05
USA-2	20,603,617	6,128,464,774	47.15	95.07
USA-3	22,691,144	6,761,304,624	47.40	95.06
Hang-1	24,099,233	7,170,632,760	46.86	95.11
Hang-2	22,126,702	6,587,239,876	47.56	95.07
Hang-3	32,326,508	9,629,968,446	46.60	92.52

**Table 2:** Statistics of assembly results.

Transcripts Length (bp)	Transcripts	Unigenes
200–300	35,479 (26.00%)	34,198 (38.28%)
300–500	23,689 (17.36%)	20,464 (22.90%)
500–1000	26,171 (19.18%)	16,520 (18.49%)
1000–2000	28,275 (20.72%)	10,600 (11.86%)
2000+	22,831 (16.73%)	7564 (8.47%)
Total Number	136,450	89,347
Total Length (bp)	147,822,489	65,896,421
N50 Length (bp)	1906	1318
Mean Length (bp)	1083	738

**Table 3:** Differentially expressed genes (DEGs) related to cell wall metabolism.

DEGs	log2FC	Regulated	Name	Gene Annotation
*c58119.graph_c1*	−1.08	down	CSLH1	Cellulose synthase-like protein H1
*c59759.graph_c0*	−8.64	down	EGL	Endoglucanase
*c40345.graph_c0*	2.11	up	EGL11	Endoglucanase 11
*c54912.graph_c1*	1.31	up	EGL24	Endoglucanase 24
*c25441.graph_c0*	1.39	up	EGL6	Endoglucanase 6
*c48168.graph_c0*	1.16	up	EGL6	Endoglucanase 6
*c51124.graph_c4*	1.82	up	EGL8	Endoglucanase 8
*c35248.graph_c0*	1.07	up	EXPA1	Expansin-A1
*c55140.graph_c2*	1.45	up	EXPA4	Expansin-A4
*c60876.graph_c0*	1.71	up	EXPB3	Expansin-B3
*c46611.graph_c0*	1.10	up	EXPB3	Expansin-B3
*c25817.graph_c0*	1.24	up	GALA2	Galactoside 2-alpha-L-fucosyltransferase
*c57127.graph_c0*	1.30	up	GATL2	Galacturonosyltransferase-like 2
*c57645.graph_c0*	−3.04	down	SPS4	Sucrose-phosphate synthase 4
*c47890.graph_c0*	−1.99	down	SUS5	Sucrose synthase 5
*c60320.graph_c1*	−8.14	down	XTH2	Xyloglucan endotransglucosylase/hydrolase 2
*c60320.graph_c0*	−7.83	down	XTH22	Xyloglucan endotransglucosylase/hydrolase protein 22
*c57140.graph_c2*	1.66	up	XTH8	Xyloglucan endotransglucosylase/hydrolase protein 8
*c58642.graph_c3*	1.46	up	XXT5	Xyloglucan glycosyltransferase 5

**Table 4:** DEGs controlling cell cycle and cell division.

DEGs	log2FC	Regulated	Gene	Gene Annotation
*c54419.graph_c0*	1.28	up	CYCD4-1	Cyclin-D4-1
*c57058.graph_c0*	−1.10	down	DCC1	sister chromatid cohesion protein
*c58714.graph_c1*	−1.25	down	CLB1	G2/mitotic-specific cyclin
*c58562.graph_c2*	8.28	up	MIP1	MND1-interacting protein 1
*c31188.graph_c0*	1.93	up	USP17L2	Ubiquitin carboxyl-terminal hydrolase 12
*c50340.graph_c0*	2.65	up	CYCU4-1	Cyclin-U4-1

**Table 5:** DEGs related to hormones involved in fruit development.

Hormone	DEGs	log2FC	Regulated	Gene Name	Annotation	References
Auxin	*c46159.graph_c0*	1.29	up	ABP20	auxin-binding protein ABP20	
Auxin	*c61117.graph_c0*	1.07	up	AX15A	auxin-induced protein 15A	
Auxin	*c25446.graph_c0*	1.40	up	AX10A	auxin-induced protein X10A	
Auxin	*c61022.graph_c0*	1.09	up	AX10A	auxin-induced protein X10A	
Auxin	*c60738.graph_c0*	−1.59	down	AX17	auxin-induced protein X17	Su et al. [45]
Auxin	*c54941.graph_c0*	1.00	up	AFB3	Protein auxin SIGNALING F-BOX 3	
Auxin	*c25539.graph_c0*	−1.45	down	PIN8	auxin efflux carrier component 8	
Cytokinin	*c51465.graph_c0*	−1.63	down	CKX1	cytokinin dehydrogenase 1	
Cytokinin	*c54736.graph_c2*	−1.25	down	CKX3	cytokinin dehydrogenase 3	Bartrina et al. [46]
Cytokinin	*c56658.graph_c1*	1.60	up	CKX7	cytokinin dehydrogenase 7	
gibberellin	*c54210.graph_c0*	−2.55	down	GA3OX1	gibberellin 3-beta-dioxygenase 1	Rieu et al. [47]

**Table 6:** Transcription factors (TF) regulating fruit size.

TFs	DEGs	log2(Fold Change)	Regulation
APRR2	*c57380.graph_c0*	1.51	up
bHLH	*c50592.graph_c0*	−1.04	down
bHLH	*c51433.graph_c0*	−5.87	down
bHLH	*c59721.graph_c1*	1.59	up
bHLH	*c49783.graph_c0*	1.22	up
bZIP	*c52788.graph_c0*	−6.03	down
bZIP	*c49079.graph_c0*	−5.71	down
ERF	*c60551.graph_c0*	1.90	up
ERF	*c26154.graph_c0*	4.02	up
ERF	*c56200.graph_c0*	2.79	up
ERF	*c38764.graph_c1*	2.29	up
ERF	*c48136.graph_c0*	5.76	up
ERF	*c41795.graph_c0*	2.43	up
ERF	*c38764.graph_c0*	1.92	up
ERF	*c48845.graph_c0*	1.81	up
MYB-like	*c46418.graph_c1*	−1.55	down
MYB-like	*c60190.graph_c8*	1.53	up
MYC-like	*c60674.graph_c0*	1.78	up
RAD	*c28120.graph_c0*	−3.00	down
RAD	*c58182.graph_c0*	−1.39	down
RAD	*c46206.graph_c0*	−2.77	down
TFIIB	*c53245.graph_c1*	1.15	up
WRKY	*c49238.graph_c0*	−4.92	down
WRKY	*c40434.graph_c0*	−1.55	down
WRKY	*c48149.graph_c0*	−1.57	down
WRKY	*c54131.graph_c0*	1.36	up

## Supplementary Materials

The following are available online at https://www.mdpi.com/2073-4425/11/4/359/s1, **Table S1:** Primer list of genes selected for qRT-PCR; **Table S2**. List of the differentially expressed genes between Hang and USA fruits; **Figure S1**. Unigene length distribution.
